# Spatiotemporal Dynamics of Pro-Inflammatory Mediator Expression in *Pelteobagrus vachelli* During Ichthyophthiriasis: A 40-Day Longitudinal Study of *IL-1β*, *IL-6*, and *SAA*

**DOI:** 10.3390/ani15111577

**Published:** 2025-05-28

**Authors:** Yang He, Miaomiao Wang, Jun Wang, Yadong Wang, Xiaolan Pi, Qi Wang, Yan Fu, Wei Fan, Qian Yang

**Affiliations:** 1 Fishes Conservation and Utilization in the Upper Reaches of the Yangtze River Key Laboratory of Sichuan Province, NeiJiang 641000, China; he_yang_yang@126.com (Y.H.); 10001407@njtc.edu.cn (J.W.); 2 College of Life Science, Nei Jiang Normal University, Neijiang 641000, China; wangmm1313@163.com (M.W.); yskez@outlook.com (Y.W.); 15328527900@163.com (X.P.); 19882483557@163.com (Q.W.); 18284899326@163.com (Y.F.); 3 School of Marine Science and Fisheries, Jiangsu Ocean University, Lianyungang 222000, China; 4 Neijiang Academy of Agricultural Sciences, Neijiang 641000, China; fanweymed@163.com; 5 Laboratory Animal Center, Southwest Medical University, Luzhou 646000, China

**Keywords:** *Ichthyophthirius multifiliis*, *Pelteobagrus vachelli*, interleukin-1β, interleukin-6, serum amyloid A

## Abstract

Interleukin-1β (IL-1β), interleukin-6 (IL-6), and serum amyloid A (SAA) are key pro-inflammatory mediators in the regulation of immune responses. The hypothesis posits that their expression varies with the progression of *Ichthyophthirius multifiliis* (Ich) infection and may serve as predictors of disease outcome. To elucidate their spatiotemporal dynamics, the full-length CDS of *IL-1β*, *IL-6*, and *SAA* in *Pelteobagrus vachelli* were cloned, and then their mRNA levels were tracked from infection onset (day 0) to resolution (day 40) or mortality in seven tissues. Key findings revealed a surge in *IL-1β* expression in the kidney and *SAA* expression in the spleen at 5 days post-infection (dpi), coinciding with no visible symptoms. Significant *SAA* elevation in the head kidney was observed at 10 dpi, preceding the emergence of white spots on the skin. This was followed by gill-specific *SAA* downregulation at 20 dpi, when 40% of fish presented with white spots. Complete clinical resolution by 40 dpi correlated with reduced hepatic *SAA* and branchial *IL-1β* levels. Notably, the concurrent upregulation of all three mediators occurred exclusively in the skin of moribund individuals. These findings identify potential biomarkers for tracking host inflammatory responses in ichthyophthiriasis.

## Simple Summary

The *Pelteobagrus vachelli* (darkbarbel catfish) has emerged as an important economic fish species in China. However, its high susceptibility to *Ichthyophthirius multifiliis* (Ich) infection poses major challenges to sustainable farming. The limited understanding of the species’ immune response to Ich has hindered the development of disease management strategies. The present investigation of the pro-inflammatory mediators IL-1β, IL-6, and SAA revealed three advances: (1) tissue-specific expression patterns are correlated with infection progression, (2) early cytokine surges precede clinical signs, and (3) terminal hyperinflammation is observed in moribund individuals. These findings provide a spatiotemporal map of inflammatory dynamics during ichthyophthiriasis, offering new methods of early diagnostics and immunomodulatory interventions in aquaculture.

## 1. Introduction

*Ichthyophthirius multifiliis* (referred to as Ich) is a highly pathogenic ciliate, causing significant economic losses to the freshwater aquaculture industry. Ich is an obligate parasite that affects nearly all freshwater teleosts, triggering outbreaks of ichthyophthiriasis [1,2]. Its parasitic lifestyle commences when a theront invades the epidermis of a freshwater fish and terminates when it further evolves into a trophont and sloughs off the host. During this period, the infected fish exhibits “white spot disease”, characterized by white spots on the surface of its skin, gills, and fins [3]. These spots impair respiration and osmoregulation, facilitate secondary infections, and ultimately lead to death [4]. Although fish that survive the initial infection generate protection against subsequent infection, control measures and therapeutic options for Ich remain limited [5].

The darkbarbel catfish (*Pelteobagrus vachelli*), the largest species of the genus Pelteobagrus, is an economically important freshwater fish in China [6]. It exhibits broad distribution and feeds on diverse sources, including zoobenthos, shrimp, and terrestrial plant matter [7]. Renowned for its flavorful meat, minimal intermuscular bone, and high nutritional value, *P. vachelli* is highly valued by consumers and the aquaculture industry [8,9]. Together with *Pelteobagrus fulvidraco*, its production reached approximately 622,000 tons in 2023 [10]. Although *P. vachelli* grows faster and attains a larger size than *P. fulvidraco*, it is more susceptible to exogenous pathogens, particularly ichthyophthiriasis (caused by Ich), when water temperatures range from 18 °C to 25 °C. Currently, Ich has emerged as one of the major bottlenecks hindering the development of the fish aquaculture industry and poses considerable economic threats [11].

Pro-inflammatory cytokines are mediators that regulate inflammation, which is a cascade of gene products usually not produced in healthy individuals [12]. Inflammatory processes are known to occur during the Ich infection. Interleukin-1 beta (IL-1β), interleukin-6 (IL-6), and serum amyloid A (SAA) are well-known pro-inflammatory cascades, exhibiting high expression levels in rainbow trout (*Oncorhynchus mykiss*) during Ich infections [13,14,15]. We hypothesized that their expression dynamics during Ich infection could predict disease progression in darkbarbel catfish. However, little is known about spatiotemporal expression profiles from Ich infection onset to clinical resolution or mortality in different tissues.

In the present study, we established a cohabitat infection model of ichthyophthiriasis in darkbarbel catfish, cloned the complete coding sequences (CDS) of IL-1β, IL-6, and SAA, and dynamically monitored their transcription levels by RT-qPCR throughout the infection period. These findings provide novel insights into the spatiotemporal dynamics of inflammatory responses during ichthyophthiriasis.

## 2. Materials and Methods

### 2.1. Ethics and Legislation

Animal handling procedures were approved by the Animal Experiment Ethics Committee of Neijiang Normal University, Neijiang, China. The minimum number of fish was employed in the experiment, and fish were euthanized with MS222 (100 mg/L) before being dissected. Furthermore, moribund fish exhibiting clinical signs, including lethargy, hyperventilation, and equilibrium disturbance, were removed from the tank and euthanized to death.

### 2.2. Fish

Darkbarbel catfish (weighing 8.0 ± 2.0 g) were purchased from a local fish farm (Neijiang, Sichuan, China). Infection-free status was confirmed by standard bacteriological, parasitological, and yellow catfish calicivirus examinations [16,17,18]. They were then acclimatized for 14 days at 20 °C in cylindrical plastic culture tanks (with a diameter of 1.0 m and a height of 1.2 m) filled with 500 L of aerated tap water.

### 2.3. Gene Cloning and Phylogenetic Analysis

Healthy fish were euthanized for RNA extraction. Following euthanasia, tissues from the spleen, liver, kidney, and heart were collected, pooled, and homogenized for RNA extraction using the Animal Total RNA Isolation Kit (Foregene Biotech, Chengdu, China). Subsequently, cDNA was synthesized according to the manufacturer’s instructions using the FastKing cDNA Kit (with gDNase) (TransGen Biotech, Beijing, China). The cDNA was used as the template for PCR to amplify the full-length CDS of *IL-1β* and *IL-6*. Moreover, to amplify the *SAA* CDS, the cDNA template was extracted from the liver of an injured fish. The fish was subjected to a 0.5 mm wound at the base of the dorsal fin using a sterile surgical blade and was sampled 4 h post-injury. Tissue homogenates underwent RNA extraction and cDNA synthesis. The cDNA template was used to amplify the *SAA* CDS. PCR primer design was performed according to the predicted sequences from the NCBI GenBank database using Oligo 6.0 (Table 1).

The PCR reaction (50 μL) was prepared as follows: 25 μL of a 2× PCR mix, 2 μL of the cDNA template, 4 μL of the primer mixture (10 μM each of the forward and reverse primers), and 19 μL of RNase-free water. The amplification conditions were initial denaturation at 94 °C for 5 min; 30 cycles of denaturation at 94 °C for 30 s, annealing at 55 °C (for *IL-1β* and *IL-6*) or 53 °C (for *SAA*) for 30 s, and extension at 72 °C for 1 min; followed by a final extension at 72 °C for 10 min. The amplified products were analyzed by 1% agarose gel electrophoresis, purified, and ligated into the pEASY-T1 cloning vector (TransGen Biotech, Beijing, China). The ligated plasmids were then transformed into *E. coli* DH5α. Positive clones were screened by culturing on LB agar plates containing ampicillin (100 μg/mL), further verified by colony PCR with gene-specific primers, and sent for sequencing (Sangon Biotech Co., Ltd., Shanghai, China). The obtained CDS sequences of *SAA*, *IL-1β,* and *IL-6* were submitted to the NCBI GenBank database. The confirmed recombinant strains were designated as DH5α-pEASY-T1-SAA, DH5α-pEASY-T1-IL-1β, and DH5α-pEASY-T1-IL-6 and stored at −80 °C in LB medium supplemented with 10% glycerol for further use.

Phylogenetic analysis was conducted, and the phylogenetic tree was built according to Huang et al. [19]. Briefly, the CDSs of additional fish species were retrieved from the NCBI GenBank database and aligned using the ClustalW algorithm implemented in MEGA (version 7.0.26). A maximum-likelihood (ML) phylogenetic tree was constructed with IQ-TREE (version 2.2.0), employing ModelFinder to select the optimal substitution model under the -m TEST parameter. Branch support was assessed with 1000 ultrafast bootstrap replicates (−bb 1000). The final tree topology was edited using Adobe Illustrator (version 25.2.3).

### 2.4. Infection

#### 2.4.1. Parasites

Ich was obtained from infected darkbarbel catfish in a local fish farm in Neijiang. Theronts were prepared, and their concentration was determined following the method of Li et al. [20]. The resulting theronts were used to infect naive fish by adding parasites to a 100 L fish tank with a concentration of 1000 theronts/fish. Infected fish were then used as donor fish in the following experiment.

#### 2.4.2. Experimental Grouping

Two experimental tanks (*n* = 30 fish/tank) were prepared, one for Ich infection exposure (infection tank) and the other as an uninfected control (control tank). Both tanks contained 500 L of aerated tap water, with 50% water replenishment after each sampling. Environment parameters were maintained at 24 °C (temperature-controlled room), <0.5 mg/L ammonia, and ≥6 mg/L dissolved oxygen through continuous airstone aeration. Fish were fed once a day with commercial feed pellets (1% biomass/d; Tongwei Group, Sichuan, China).

#### 2.4.3. Ich Challenge and Sampling

The cohabitation infection experiment was conducted as described by Bonnichsen et al. [21], with minor modifications. Briefly, three Ich-infected fish carrying >50 white spots were marked via caudal fin clipping (removing 20% of the total fin area) and cohabited with healthy fish in the infection tank, whereas three uninfected fish (sham controls) were added as negative controls. All donor fish were removed after 48 h of exposure.

Following the challenge, fish were monitored daily. Ten fish per tank were randomly collected and transferred to a 500 mL aerated tap-water-filled transparent plastic storage box for white spot observation. At each sample time point, namely 5 days post-infection (dpi; no clinical signs), 10 dpi (100% prevalence), 20 dpi (40% prevalence), and 40 dpi (complete resolution), five individuals were randomly euthanized for tissue collection (liver, spleen, kidney, head kidney, gills, and skin). Tissues were preserved in RNAstore (Tiangen Biotech, Beijing, China). Moribund specimens were similarly processed and recorded as BS. No clinical signs were observed in control tanks. Terminal sampling at 40 dpi included five control fish (designated as the 0 dpi non-infected group). Microscopic and histopathological confirmations were performed following standard diagnostic protocols [22].

### 2.5. Quantitative Reverse Transcription PCR (RT-qPCR)

Primers for RT-qPCR targeting *SAA*, *IL-1β*, and *IL-6* were designed using Primer Premier^®^ 5.0 software based on the respective nucleotide sequences of target genes (Table 1). In addition, *β-actin* was utilized as the housekeeping gene for cDNA normalization. Amplification efficiency and primer specificity were determined using a standard cure separately generated from plasmid dilutions, with gradient concentrations and melt curve analysis post-amplification.

RNAstore-fixed tissues were subjected to RNA extraction. A total of 1000 ng of RNA was converted to cDNA, and the resultant cDNA was diluted 10-fold with DNase/RNase-free water to a working concentration. Gene expression profiling was performed on a CFX connectTM Real-Time PCR System (Bio-Rad, Shanghai, China). Briefly, the sample setup (10 μL) was composed of 5 μL of the 2×TransStart Top/Tip Green qPCR supermix, 1 μL of cDNA, 0.2 μL of each primer (10 μM forward primer and 10 μM reverse primer), and 3.6 μL of RNase-free water. The cycling conditions were as follows: 94 °C for 5 min, followed by 40 cycles of 94 °C for 5 s, and 60 °C (IL-1β and IL-6)/63 °C (SAA) for 30 s. Three technical replicates were run for each sample. Non-template controls and samples negative for the reverse transcriptase enzyme were included.

The expression level of SAA, IL-1β, and IL-6 was calculated using the delta-delta method (2^−ΔΔCT^) [23]. *β-actin* was used as an internal calibrator. All data are presented in terms of relative mRNA, and they are expressed as means ± standard errors (SEs).

### 2.6. Statistical Analysis

Statistical analysis and data visualization were performed using GraphPad Prism 8.0. Normality and lognormality assumptions were assessed through Shapiro–Wilk tests. Non-normally distributed data were analyzed using the Kruskal–Wallis test followed by Dunn’s post hoc test. Effect sizes for significant differences were calculated using Cohen’s d, with 95% confidence intervals. Gene expression levels were evaluated by one-way ANOVA, and multiple comparisons against the 0 dpi non-infected group were conducted using Dunnett’s method. *p*-values < 0.05 were considered to represent statistical significance.

## 3. Results

### 3.1. CDS Cloning and Phylogenetic Analysis

**Figure 1:**
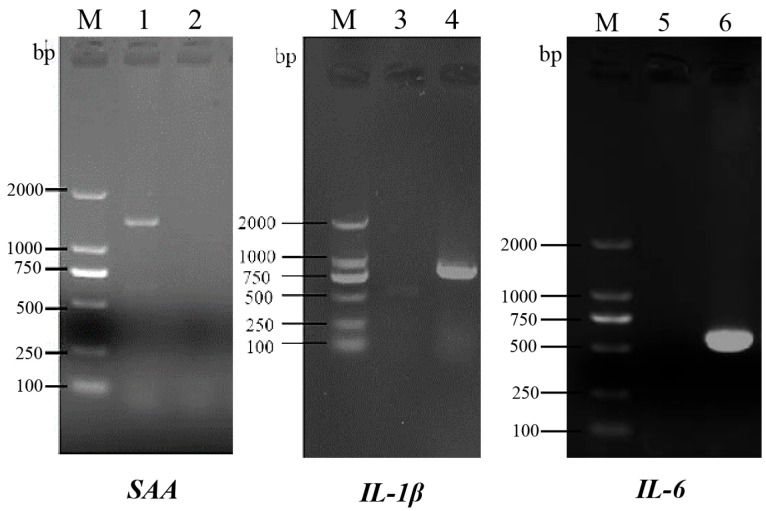
PCR amplification products of *SAA*, *IL-6*, and *IL-1β* of darkbarbel catfish. Lane M, DNA marker; lane 2, lane 3, and lane 5, negative control; lane 1, positive amplification of SAA; lane 4, positive amplification of IL-6; lane 6, positive amplification of IL-1β.

**Figure 2:**
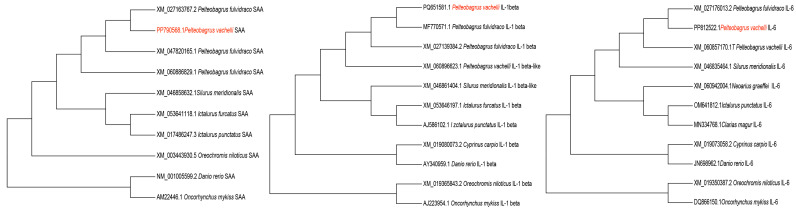
Phylogenetic trees of *SAA*, *IL-1β*, and *IL-6*. Red font represent the present GenBank ID of present study.

The full-length CDSs of *SAA* (1404 bp), *IL-1β* (729 bp), and *IL-6* (597 bp) were amplified (Figure 1) and deposited in the NCBI GenBank database, with the assigned accession numbers PP790568.1, PP812522, and PQ651581, respectively. Phylogenetic analysis showed that *SAA*, *IL-1β,* and *IL-6* had 99.5%, 98%, and 100% similarity, respectively, with *Tachysurus fulvidraco* and were clustered into one branch (Figure 2).

### 3.2. Symptoms of Infected Fish

**Figure 3:**
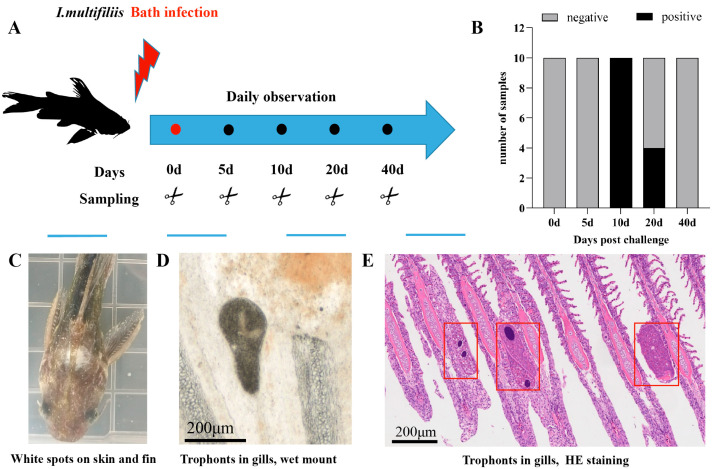
Clinical and histopathological *symptoms* of dying darkbarbel catfish. (**A**). Schedule of sampling. (**B**). Morbidity of fish post-infection. (**C**). Moribund fish infected by Ich. (**D**). Microscopic observation of gills, wet mount. (**E**). Histopathological observation of gills. Red box Trophonts, HE staining.

During the initial infection phase (0–5 dpi), all fish exhibited normal behavior with no visible white spots on the skin or fins. By 10 dpi, 100% of the cohort exhibited characteristic white spots on the skin, fins, and gills. A progressive reduction in the number of white spots became evident by 20 dpi, with only 40% of individuals presenting with white spots. Complete clinical resolution was achieved by 40 dpi, with no white spots detected (Figure 3B).

Moribund fish displayed characteristic disease progression marked by lethargy, anorexia, abnormal surface orientation, and coalescing white spots forming a generalized body coating (Figure 3C). Histopathological examination confirmed parasitic trophonts colonizing the secondary lamellae of affected gills (Figure 3D), providing definitive confirmation of Ich infection.

### 3.3. Expression of SAA, IL-1β, and IL-6

**Figure 4:**
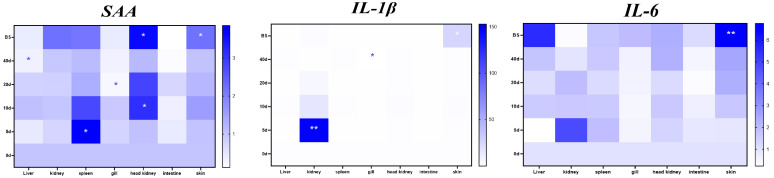
Heat map showing the relative gene expression in seven tissues at different time points. Infected fish compared to control fish. *: significant differences from control fish, 0.01 < *p* < 0.05. **: Obvious significant differences from control fish, *p* < 0.01. The hierarchical clustering orders the rows based on similarity.

**Figure 5:**
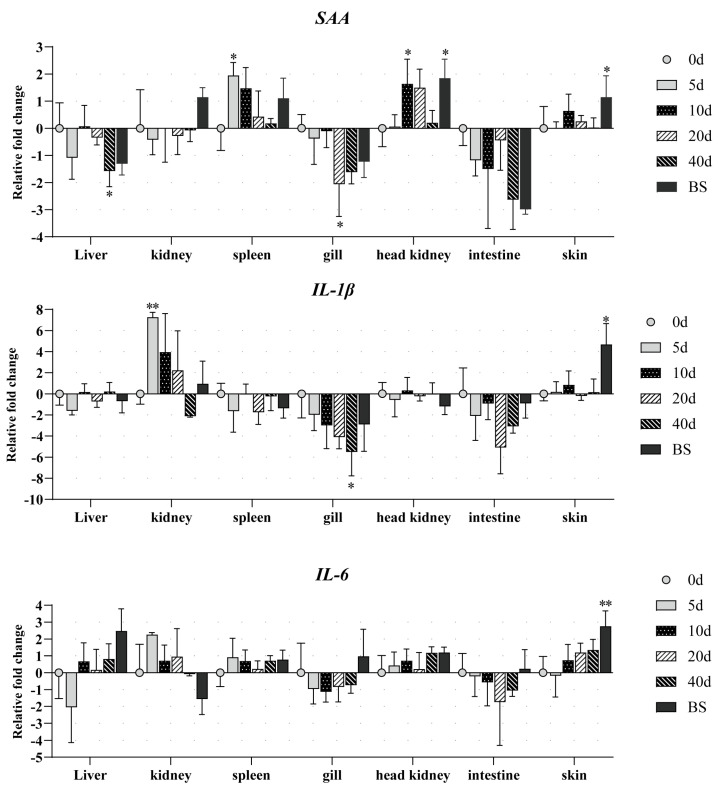
The expression of *SAA*, *IL*-*6*, and *IL*-*1β* genes after infection with Ich at various time points (0, 5, 10, 20, and 40 dpi and moribund fish). Bars and error lines represent geometrical means and geometrical standard deviations. The expression levels between infected time points and uninfected time points are significantly different (0.01 < *p* < 0.05), where the symbol * over the bars represents significant differences and ** over the bars represents extremely significant differences (*p* < 0.01).

All RT-qPCR primers exhibited amplification efficiencies within 90–110%, and their melt curves displayed single peaks (Supplementary Figure S1). The tissue-specific expression profiles of SAA, IL-1β, and IL-6 exhibited distinct dynamic patterns during Ich infection in darkbarbel catfish, as summarized in Figure 4 and Figure 5.

- *SAA*. Significant upregulation of SAA was observed in the spleen, head kidney, and skin. In the spleen, SAA exhibited the earliest expression surge, with a 4-fold increase compared to uninfected controls at 5 dpi, followed by a gradual decline. The head kidney displayed parallel kinetics to the spleen, with SAA levels peaking at 10 dpi (3-fold increase). Notably, moribund fish showed a further elevation to 3.9-fold. Upregulation was observed in skin during the terminal stages, reaching 2.5-fold in moribund specimens. In contrast, downregulation patterns were detected in the liver, gills, intestine, and kidney. Significant suppression emerged in gills by 20 dpi and in the liver by 40 dpi. No statistically significant changes were observed in the intestine or kidney throughout the observation period.
- *IL-1β*. Significant upregulation of IL-1β was observed in the kidney and skin. IL-1β expression peaked at 5 dpi with a 150-fold increase compared to uninfected controls, followed by a rapid return to baseline levels. The skin of moribund fish exhibited marked induction (52.8-fold elevation vs. control fish). Pronounced downregulation emerged at 40 dpi in the gills, showing 15.4-fold suppression relative to uninfected darkbarbel catfish.
- *IL-6*. Significant changes were only found in the skin of moribund fish, showing a 7.8-fold increase compared to the uninfected controls.

## 4. Discussion

The inflammatory response is a crucial immune defense mechanism, ensuring survival during infections and maintaining homeostasis. However, excessive inflammation can exacerbate pathological processes, suggesting that pro-inflammatory cytokines may exhibit dual roles in Ich infection [24]. Although previous studies have characterized the transcription of *SAA*, *IL-1β*, and *IL-6* during Ich infection, their kinetics throughout the complete infection–recovery continuum remain uncharacterized. To address this gap, we established a sublethal Ich infection model via a cohabitation challenge and dynamically monitored the transcriptional profiles of these three genes over a 40-day period spanning from initial infection to complete recovery. To better replicate natural infection dynamics, this study modified the methodology of Jaafar et al. [25] by exposing fish to infective theronts. Our cohabitation challenge model yielded results consistent with Xiong et al. [26], showing visible white spots emerging in *P. vachelli* after 5 dpi.

SAA, a highly conserved apolipoprotein, plays a crucial role during inflammatory responses [27]. While primarily synthesized by hepatocytes, its extrahepatic synthesis has been reported in the spleen, head kidney, gills, intestine, and skin [28]. SAA transcription responds to various inducers, including IL-1β and IL-6 [29]. In the present study, *SAA* amplification was undetectable in healthy fish but successful when using cDNA templates from specimens sampled 4 h post-injury, consistent with results in Arctic char (*Salvelinris aipinus*) [30]. These results suggest that SAA may serve as a potential biomarker for monitoring *P. vachelli* health status, aligning with prior reports [31]. In Atlantic salmon (*Salmo salar* L.), spleen SAA exhibited upregulation on days 1, 5, and 15 after infection with the salmon louse (*Lepeophtheirus salmonis Krøyer*), while downregulation was observed on days 3 and 10 [32]. Similarly, SAA upregulation has been documented in common carp (*Cyprinus carpio*) during inflammatory responses to turpentine oil and *Aeromonas hydrophila* [33,34]. In the present study, significant *SAA* upregulation was found in the spleen at 5 dpi and the head kidney at 10 dpi. A previous study reported that the skin of the common carp is a major site of SAA production during the acute-phase response to Ich infection, with expression peaking at 1600-fold 36 h post-infection [35]. However, no significant SAA transcriptional changes were detected in the skin in our study, except in moribund individuals. Notably, marked downregulation of SAA occurred in the gills (20 dpi) and livers (40 dpi). This discrepancy may stem from differences in sampling time points, as SAA is likely triggered during the early infection stages. Our sampling intervals were designed to capture the overall infection–recovery trajectory rather than acute-phase-specific dynamics. The early elevated SAA in the spleen and head kidney suggests the rapid activation of the innate immune response, whereas the reduced SAA in the liver, gills, and intestine may imply the host’s recovery from Ich infection.

Previous studies in trout macrophages demonstrated that IL-1β induces inflammatory responses and drives acute-phase reactions [36]. For example, IL-1β upregulation was observed in rainbow trout larvae as early as 3 h after Ich infection [14] (P2). Similarly, SAA upregulation was detected in zebrafish (*Danio rerio*) gills at 4 dpi [37]. In rainbow trout, IL-1β upregulation has been documented in the skin (4, 6, and 26 dpi), head kidney (24 h post-infection), hip (6 dpi), and spleen (24 hpi and 26 dpi) [13] (p2). In contrast, our study revealed *IL-1β* upregulation only in the kidneys at 5 dpi and the skin of moribund fish, alongside a marked downregulation in the gills at 40 dpi. This discrepancy may reflect the host’s recovery from infection. IL-1β is the key inflammatory cytokine of the IL-1 family, serving as an “alarmin” due to its role in alerting the host to initiate immune responses [38]. The tissue-specific dynamic implies compartmentalized immune regulation during early infection.

IL-6 is a multifunctional cytokine involved in the regulation of acute-phase reactions, inflammatory responses, and the transition from innate to adaptive immunity [39]. Studies have demonstrated that IL-6 overproduction may trigger a cytokine storm, leading to systemic hyperinflammation [40]. Notably, tissue-specific inhibition of IL-6 signaling has been shown to significantly attenuate cytokine-storm-induced tissue damage [41]. In our study, *IL-6* mRNA expression levels in all examined tissues remained unaltered by Ich infection, except in the skin of moribund fish. This suggests that suppression of IL-6 signaling might mitigate Ich-induced tissue injury. In contrast, others have reported the essential role of IL-6 in protective immune responses in the skin against *Litomosoides sigmodontis* in mice [42]. However, while therapy targeting IL-6 is broadly used in chronic and acute inflammatory diseases, whether this therapy is effective or only partially effective still requires validation.

In the present experiment, the expression levels of the three genes mirrored a similar downward trend in both gills and intestines, although most of these differences did not reach statistical significance when compared to the control group. The differential expression of *SAA*, *IL-1β*, and *IL-6* was statistically nonsignificant at most time points following Ich infection, suggesting that these molecules may primarily be activated during the acute phase of infection. Notably, significant upregulations were observed in the spleen (*SAA*) and kidney (*IL-1β*) at 5 dpi, indicating the occurrence of inflammatory responses during this period. Therefore, *SAA* in the spleen and *IL-1β* in the kidney could serve as potential early indicators of Ich infection, as they were significantly upregulated prior to the appearance of typical clinical signs (e.g., white spots). Furthermore, significant upregulation of all three genes was observed in the skin of moribund fish. We hypothesize that the inflammatory reaction might be exacerbated by secondary infections, potentially hindering the healing process in Ich-infected fish. However, further research is required to validate this hypothesis.

## 5. Conclusions

This study delineated the spatiotemporal dynamics of *SAA*, *IL-1β*, and *IL-6* gene expression during Ich infection. The results demonstrated tissue-specific patterns: *SAA* was upregulated in the spleen, head kidney, and skin at 5 and 10 dpi, but downregulated in the gills (20 dpi) and the liver (40 dpi). *IL-1β* showed early upregulation at 5 dpi and late downregulation at 40 dpi. Notably, moribund fish exhibited concurrent upregulation of all three mediators exclusively in the skin. These results advance the understanding of Ich pathogenesis and host inflammatory responses.

## Figures and Tables

**Table 1:** Primers used in the present research.

Primer	Primer Sequence (5′-3′)	GenBank Accession No.	Length (bp)	Experiment
*IL-1β* F	ATGGCTGGCGAAGATTTT	XM_060895816.1	729	Gene cloning
*IL-1β* R	TCAGAACTCATTCTGAGAGACTACT			
*q-IL-1β* F	ACCAGGACCTCTTCACTATCT	PQ651581	98	RT-qPCR
*q-IL-1β* R	GTCCTGCATGCTGTAACTCT			
*IL-6* F	ATGGATTTCTATGAAACATCTGGA	XM_027176013.2	597	Gene cloning
*IL-6* R	TCATTGCTGGTGTTTTGAGATCCA			
*q-IL-6* F	AGATGCCGATCCTCAACAAT	PP812522	100	RT-qPCR
*q-IL-6* R	ACCTGGTACACCCGCAAACC			
*SAA* F	ATGTTTATGGACTCACCACAGATG	XM_060886829.1	1404	Gene cloning
*SAA* R	TTACACTGGCTCTGGTTTAGGCTC			
*SAA* F	GTACAGCAGCCTCCAGT	PP790568	111	RT-qPCR
*SAA* R	ATGAGGAATTGATGAAGAGC			
*β-actin* F	GATTCGCTGGAGATGATGCT	EU161066.1	162	RT-qPCR
*β-actin* R	CGTGCTCAATGGGGTACTTC			

## Data Availability

The original contributions presented in this study are included in the article/Supplementary Material. Further inquiries can be directed to the corresponding author.

## Supplementary Materials

The following supporting information can be downloaded at: https://www.mdpi.com/article/10.3390/ani15111577/s1. Figure S1. The standard Curve and melt peak of SAA, E = 96.3%. Figure S2. The standard Curve and melt peak of IL-1β, E = 106.3%. Figure S3. The standard Curve and melt peak of IL-6, E = 90.7%.

## Abbreviations

The following abbreviations are used in this manuscript:

Ich	*Ichthyophthirius multifiliis*
PCR	Polymerase Chain Reaction
SAA	Serum Amyloid A
IL-1β	Interleukin-1 beta
IL-6	Interleukin-6
