# Supplementary material for: Spatiotemporal Dynamics of Pro-Inflammatory Mediator Expression in Pelteobagrus vachelli During Ichthyophthiriasis: A 40-Day Longitudinal Study of IL-1β, IL-6, and SAA
Source: Animals (Basel). 2025 May 28;15(11):1577. doi: 10.3390/ani15111577 (PMC12153630; doi:10.3390/ani15111577)
Supplement: Supplementary file 1 [file animals-15-01577-s001.zip › animals-3621793-supplementary.pdf]

# Supplementary 1

The amplification efficiency of the RT-qPCR primers was validated using standard curves and melt curve analysis confirmed the specificity of amplification. Results showed that all RT-qPCR primers exhibited amplification efficiencies within 90-110% and their melt curves displayed single peals (figure S1-3）.

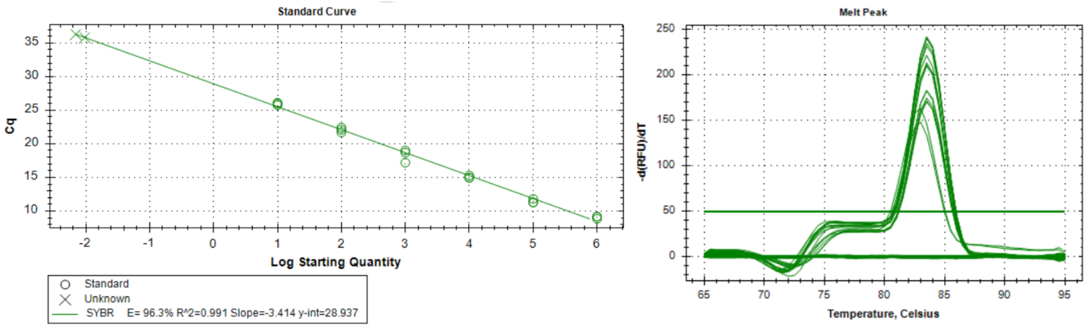


Figure S1. The standard Curve and melt peak of SAA, E=96.3%

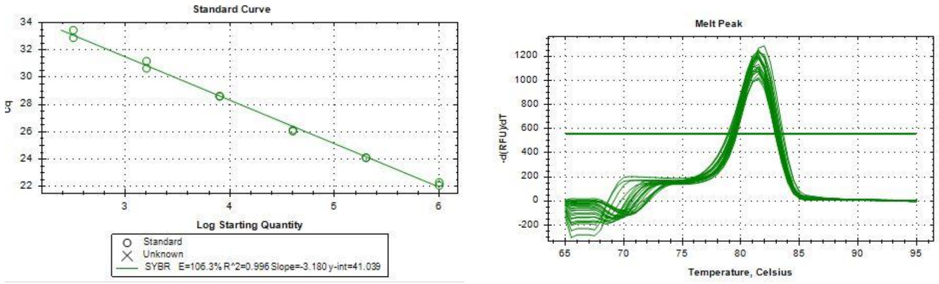


Figure S2. The standard Curve and melt peak of IL-1β，E=106.3%

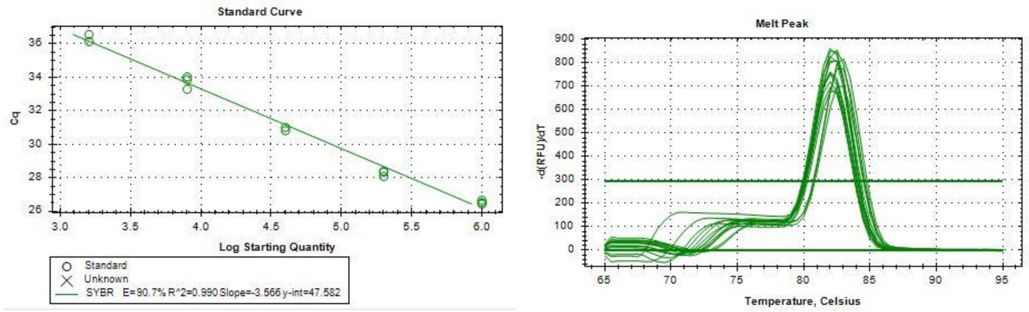


Figure S3. The standard Curve and melt peak of IL-6, E=90.7%
